# Supplementary material for: Platinum(IV) Complexes of the 1,3,5-Triamino Analogue of the Biomolecule Cis-Inositol Designed as Innovative Antineoplastic Drug Candidates
Source: Pharmaceutics. 2022 Sep 27;14(10):2057. doi: 10.3390/pharmaceutics14102057 (PMC9611922; doi:10.3390/pharmaceutics14102057)
Supplement: Supplementary file 1 [file pharmaceutics-14-02057-s001.zip › pharmaceutics-1842994-supplementary.pdf]

Platinum(IV) complexes of the 1,3,5-triamino analogue of the biomolecule cis-inositol designed as innovative antitumor drugs' candidates

Vyara Velcheva [1], Kaspar Hegetschweiler [2], Georgi Momekov [3,*], Stefka Ivanova [3,4], Angel Ugrinov [4], Bernd Morgenstern [2] and Galina Gencheva [1,*]

[1] Faculty of Chemistry and Pharmacy, Sofia University "St. Kliment Ohridski", 1 J. Bourchier Blvd., 1164 Sofia, Bulgaria; ahvnv@chem.uni-sofia.bg (VV, 0000-0002-1476-7224); (GG, 0000-0003-1696-1589)

[2] Fachrichtung Chemie, Universität des Saarlandes, Campus, D-66123 Saarbrücken, Germany; hegetschweiler@mx.uni-saarland.de (KH, 000-0001-9094-9794); b.morgenstern@mx.uni-saarland.de (BM, 0000-0002-8360-4218)

[3] Department of Pharmacology, Pharmacotherapy and Toxicology, Faculty of Pharmacy, Medical University of Sofia, 2 Dunav Str., 1000 Sofia, Bulgaria; (GM, 0000-0003-2841-7089)

[4] Department of Pharmaceutical Chemistry and Pharmacognosy, Faculty of Pharmacy, Medical University of Pleven, 1 St. Kliment Ohridski Str., 5800 Pleven, Bulgaria; ivanovastefka_pharm@yahoo.com

[5] Department of Chemistry and Biochemistry, North Dakota State University, 1311 Albrecht Blvd., Fargo, ND 58102, USA; angel.ugrinov@ndsu.edu (0000-0002-4284-6923)

* Correspondence: gmomekov@pharmfac.mu-sofia.bg (GM); ggencheva@chem.uni-sofia.bg (GG)

Electronic supplementary information

Content

1. **X-Ray analysis data** 3

1.1. **Table S1.** Selected crystallographic parameters for **1A**, **1B**, **1C** and **2A**. 3

1.2. **Table S2.** Selected bond lengths [Å] and bond angles [°] for **1A**, **1B**, **1C** and **2A** with estimated standard deviations in parentheses. 4

1.3. **Table S3.** Bond lengths [Å] and bond angles [°] of hydrogen bonds. 5

1.4. **Figure S1.** Packing of the molecules within the unit cell down the a-axis: (a) **taciplatin**, $[PtI_3(taci)]I \cdot 3H_2O$, **1A**; (b) $[PtI_3(taci)]I$, **1B**; (c) $[PtI_3(taci)]I \cdot 2DMF$, **1C**; and down the b-axis (d) **ditaciplatin**, $[Pt(taci)_2](CO_3)_2 \cdot 6H_2O$, **2A**. 6

1.5. **Figure S2.** Structure of the complexes **1B** (a) and **1C** (b). 6

1.6. **Figure S3.** Projection along the plane determined of O1S, Pt, C8, O3S in **2A**. 7

2. **FTIR spectra** 7

2.1. **Table S4**. Selected IR spectral data. 7

3. **Solid State NMR spectra** 8

3.1. **Figure S4.** ^{15}N CP-MAS NMR spectrum of **1A.** 9

3.2. **Figure S5.** ^{15}N CP-MAS NMR spectrum of **2A.** 9

3.3. Figure S6. ^{13}CP-MAS NMR spectrum of **1A.** 10

3.4. Figure S7. ^{13}C CP-MAS NMR spectrum of **2A.** 10

4. NMR Spectra (in solution) 11

4.1. Figure S8. ^{13}C-NMR spectrum of taci·2H_2O in D_2O, pD=8.4. 11

4.2. Figure S9. ^{13}C-NMR spectrum of **1** (a) and **2** (b) in D_2O. 11

1. X-Ray analysis data

Table S1. Selected crystallographic parameters for **1A**, **1B**, **1C** and **2A**.

Identification code	**1A**	**1B**	**1C**	**2A**
Empirical formula	$C_6H_{15}N_6O_3PtI_4 \cdot 1.9H_2O$	$C_6H_{15}N_3O_3PtI_4$	$C_6H_{15}N_3O_3Pt\ I_4 \cdot 2(C_6H_7NO)$	$C_{12}H_{30}N_6O_6Pt \cdot 2CO_3 \cdot 6H_2O$
Formula weight	914.42	879.90	1026.09	776.62
Crystal system	monoclinic	triclinic	monoclinic	monoclinic
Space group, **Z**	$P2_1/c$, **8** (no. 14)	P-1, **2** (no. 2)	$P2_1/n$, **4** (no. 14)	C2/c, **4** (no. 15)
a[Å]	14.7525(17)	8.310(2)	8.5050(13)	18.2792(16)
b[Å]	14.7601(18)	9.679(3)	14.441(3)	9.4678(8)
c[Å]	18.3408(19)	10.064(3)	20.993(4)	14.6798(12)
α[°]	90	94.437(9)	90	90
β[°]	108.102(4)	91.623(8)	93.956(6)	103.176(2)
γ[°]	90	96.250(9)	90	90
Volume[$Å^3$]	3796.0(8)	801.7(4)	2572.2(8)	2473.7(4)
ϱ_{calc}[g/cm^3]	3.200	3.645	2.650	2.088
μ [mm^{-1}]	13.913	16.454	10.285	5.771
Tmin,Tmax	0.0648 , 0.1495	0.0299, 0.0911	0.0037, 0.0183	0.2787, 0.7456
F(000)	3241	772.0	1864.0	1560.0
Radiation	MoKα (λ = 0.71073)	MoKα (λ = 0.71073)	MoKα (λ = 0.71073)	MoKα (λ = 0.71073)
2Θ range for data collection[°]	4.158 to 52.822	4.062 to 50.164	3.426 to 50.032	4.874 to 55.044
Total Reflections, R_{int} [%]	40445, 8.2	7477, 6.66	15905, 9.57	13768, 6.52
Indep.Refl., Refl.[I>=2σ]	7736, 6397	2793, 2352	4467 , 3136	2840, 2109
Goodness-of-fit on F^2	1.043	1.041	1.001	0.957
R1 and wR2 [I>=2σ] [%]	3.64, 7.69	4.07, 10.17	5.35, 13.49	2.41, 6.03
R1 and wR2 [%]	4.85, 8.07	5.12, 10.63	7.87,15.11	3.37, 6,81

| - |Fc|| / Σ|Fo|, $wR_2 = [\Sigma w[(Fo)^2 – (Fc)^2]^2 / \Sigma w(Fo^2)^2]^{1/2}$ for $Fo^2 > 2\sigma(Fo^2)$, $w = [\sigma^2(Fo)^2 + (AP)^2 + BP]^{-1}$ where $P = [(Fo)^2 + 2(Fc)^2] / 3$

Table S2. Selected bond lengths [Å] and bond angles [°] for **1A**, **1B**, **1C** and **2A** with estimated standard deviations in parentheses.

**Compound**	**1A-A**	**1A-B**	**1B**	**1C**
**Bond distance [Å]**				
Pt1-I1	2.6367(6)	2.6373(7)	2.6461(10)	2.6181(12)
Pt1-I2	2.6376(6)	2.6380(7)	2.6448(10)	2.6395(12)
Pt1 -I3	2.6458(7)	2.6423(7)	2.6397(11)	2.6413(11)
Pt1-N1	2.108(6)	2.118(6)	2.104(9)	2.0910(11)
Pt1-N2	2.106(6)	2.127(6)	2.100(10)	2.1020(10)
Pt1-N3	2.090(6)	2.092(6)	2.110(9)	2.1220(11)
**Bond angles [°]**				
I1-Pt1-I3	91.51(2)	91.53(2)	89.20(3)	90.40(4)
I1-Pt1-I2	90.25(2)	90.86(2)	95.40(4)	92.27(4)
I2-Pt1-I3	90.88(2)	91.54(2)	89.05(3)	90.73(4)
N1-Pt1-I1	178.13(18)	178.80(17)	176.2(2)	177.6(3)
N1 -Pt1-I3	88.92(18)	89.64(18)	90.8(3)	91.9(3)
N1-Pt1-I2	91.57(18)	89.39(17)	88.4(2)	88.2(3)
N1-Pt1-N3	90.1(3)	89.8(2)	90.2(4)	87.6(4)
N2-Pt1-I1	89.12(17)	90.32(17)	86.9(3)	87.9(3)
N2-Pt1-I3	89.17(17)	89.13(18)	91.9(3)	89.0(3)
N2-Pt1-I2	179.37(17)	178.64(17)	177.5(3)	179.7(3)
N2-Pt1-N1	89.1(2)	89.4(2)	89.3(4)	91.6(4)
N3-Pt1-N2	89.6(2)	89.6(2)	89.8(4)	89.5(4)
N3-Pt1-I1	89.47(17)	89.02(17)	89.9(3)	90.1(3)
N3-Pt1-I3	178.44(18)	178.60(17)	178.1(2)	178.4(3)
N3-Pt1-I2	90.33(17)	89.74(17)	89.4(3)	90.8(3)

**Compound**	**2A**
**Bond lengths [Å]**	
Pt1-N1A	2.0684(3)
Pt1-N2A	2.0767(2)
Pt1-N3A	2.0702(3)
Pt1-N1B	2.0684(3)
Pt1-N2B	2.0767(2)
Pt1-N3B	2.0702(3)
**Bond angles [°]**	
N3B-Pt1-N2B	88.80(14)
N3B-Pt1-N1B	91.51(11)
N3B-Pt1-N1A	88.49(11)
N3B-Pt1-N2A	91.20(14)
N1B-Pt1-N2B	89.91(11)
N1A-Pt1-N1B	180.0
N2A-Pt1-N2B	180.0
N2A-Pt1-N1B	90.09(11)
N2A-Pt1-N1A	89.91(11)
N3A-Pt1-N2B	91.20(14)
N3A-Pt1-N3B	180.0
N3A-Pt1-N1B	88.49(11)
N3A-Pt1-N1A	91.51(11)
N3A-Pt1-N2A	88.80(14)
N1A-Pt1-N2B	90.09(11)

Table S3. Bond lengths [Å] and bond angles [°] of hydrogen bonds.

D	H	A	d(D-H)/Å	d(H-A)/Å	d(D-A)/Å	D-H-A/°
				**1A**		
O2A	H2A	O2S	0.82	1.98	2.762(9)	159.2
O2A	H2A	O5S	0.82	2.78	3.30(2)	122.6
N2A	H2AA	O5S[1]	0.89	2.06	2.875(19)	151.5
N2A	H2AB	O2A[1]	0.89	2.32	3.091(8)	145.2
N1B	H1BA	O3B[2]	0.89	2.32	3.097(8)	145.3
N1B	H1BB	O4S[2]	0.89	2.03	2.856(16)	152.9
O3A	H3A	O1S	0.87	2.57	3.142(11)	124.6
N1A	H1AA	O2S[3]	0.89	2.31	3.166(10)	161.2
N3A	H3AB	O1S	0.89	2.27	3.114(10)	157.9
O1S	H1SA	O3S	0.85	1.99	2.838(11)	173.4
O1S	H1SB	O4S	0.85	2.12	2.82(2)	139.2
O2S	H2SA	O5S	0.95	2.04	2.84(2)	139.7
O2S	H2SB	O3S[4]	0.95	1.98	2.856(12)	152.5
O4S	H4SA	O1A[4]	0.85	2.39	2.936(18)	122.5
O4S	H4SB	O3B	0.85	2.75	3.278(17)	121.9
O5S	H5SB	O1B[5]	0.85	2.25	2.91(2)	133.5

Symmetry codes:
[1]2-X,1-Y,-Z; [2]3-X,1-Y,1-Z; [3]2-X,1/2+Y,1/2-Z; [4]2-X,-1/2+Y,1/2-Z; [5]3-X,-1/2+Y,1/2-Z

				**1C**		
O2	H2	O2S	0.84	1.85	2.627(15)	154.2
O3	H3	O1S	0.84	1.93	2.695(15)	151.6
N1	H1B	O2S[1]	0.91	2.22	2.885(16)	129.4

Symmetry Code:
[1]1/2-X,1/2+Y,1/2-Z

				**2A**		
O1S	H1SA	O6[1]	0.85	1.94	2.760(4)	162.3
O2	H2	O8[2]	0.82	2.02	2.696(7)	139.6
O2	H2	O10[3]	0.82	1.70	2.511(6)	167.3
N2	H2B	O9[4]	0.89	1.96	2.830(7)	166.3
N2	H2B	O10[5]	0.89	1.79	2.566(6)	144.9
O2S	H2SA	O8[6]	0.71	2.11	2.752(7)	152.0
O2S	H2SA	O9	0.71	2.17	2.869(7)	171.8
O3S	H3SB	O4[7]	0.85	1.84	2.665(6)	163.3
O1	H1	O4[4]	0.82	1.95	2.634(9)	140.4
O1	H1	O4[8]	0.82	1.89	2.638(8)	151.2
O3	H3	O6	0.82	1.80	2.621(4)	174.9
N1	H1B	O1S	0.89	1.97	2.841(4)	164.9
N3	H3A	O8	0.89	2.04	2.854(8)	152.0
N3	H3A	O9[6]	0.89	1.89	2.728(6)	156.6

Symmetry codes:
[1]-X,2-Y,2-Z; [2]1/2-X,-1/2+Y,3/2-Z; [3]+X,2-Y,-1/2+Z; [4]+X,-1+Y,+Z; [5]1/2-X,3/2-Y,2-Z; [6]1/2-X,5/2-Y,2-Z; [7]1/2+X,-1/2+Y,+Z; [8]-X,-1+Y,3/2-Z

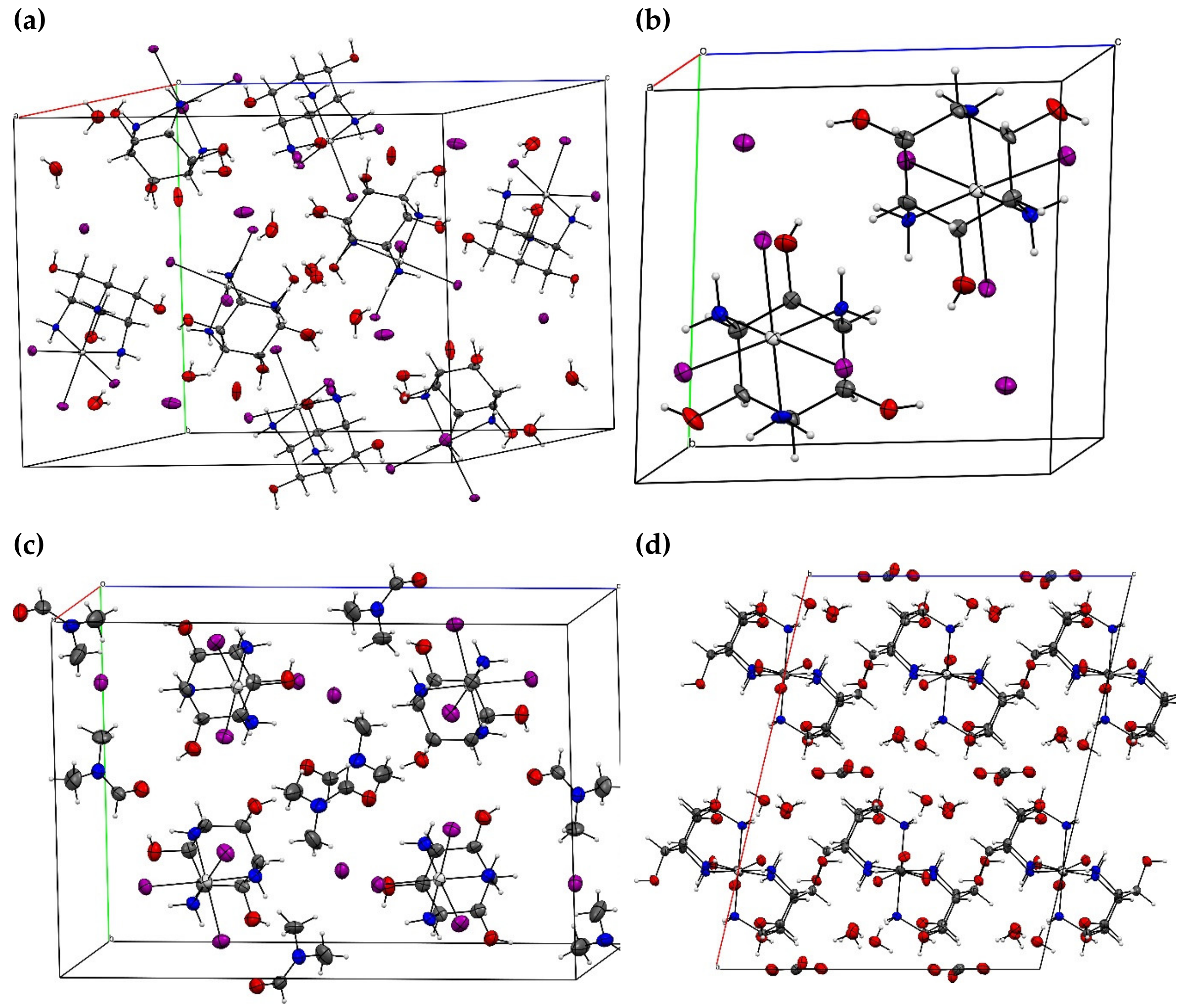

Figure S1. Packing of the molecules within the unit cell down the a-axis: (a) **taciplatin**, $[PtI_3(taci)]I\cdot 3H_2O$, **1A**; (b) $[PtI_3(taci)]I$, **1B**; (c) $[PtI_3(taci)]I\cdot 2DMF$, **1C**; and down the b-axis (d) **ditaciplatin**, $[Pt(taci)_2](CO_3)_2\cdot 6H_2O$**, 2A**.

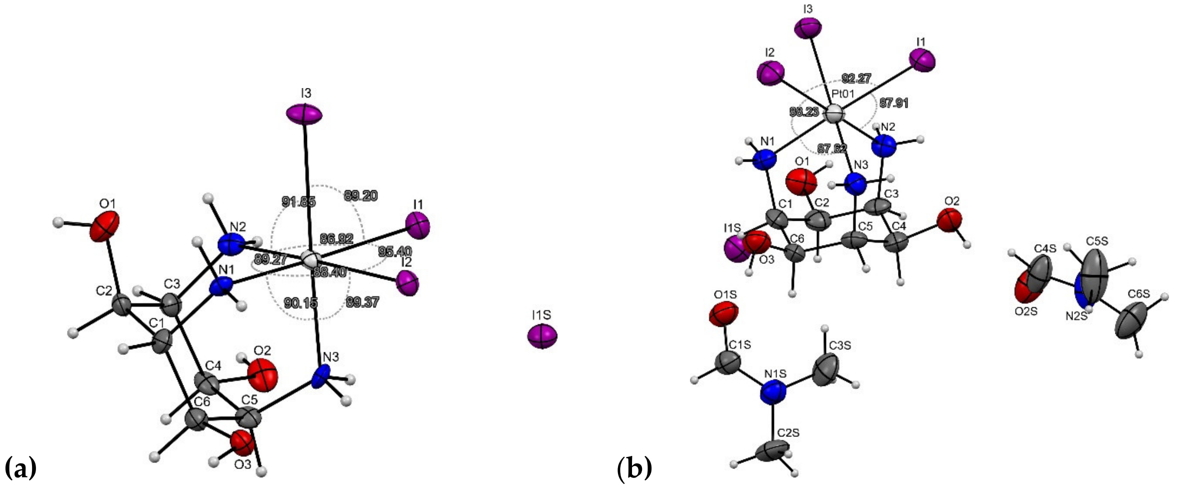

Figure S2. Structure of the complexes **1B** (a) and **1C** (b).

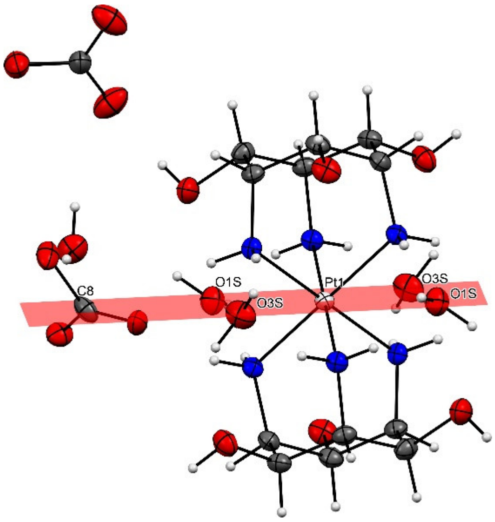

Figure S3. Projection along the plane determined of O1S, Pt, C8, O3S in the structure of **2A**.

2. FTIR spectra

Table S4. Selected IR spectral data.

Assignments/ [cm^{-1}]	taci.2H_2O	1A	2A
ν ^{as,s} (H_2O) overlapped	~3550 sh	~3580 w. br	~3510 sh.
	~3410 sh	~3530	
δ(H_2O)	1638	1620	~1640 sh.
	1620 sh		
ν(OH)	3497 str	3567	3425
	3268	3483	3357
	3180 br	3358	3324
δ(H-C-O-H) $_{\text{in plane}}$	1460 sh	1430 w	1473
	1437	1412 w	1394
	1376	1369 str	1372
ν(O-C-C-N)	1109	1102	1095 sh.
	1071	1079	1073 str
	1050	1053	
		1046	
δ(C-O-H)$_{\text{out of plane}}$	676		
	649 w		
	626		
	603 w	577	599

$\nu^{as, s}(NH_2)$	3332	3248	3262
	3259	3225	3221
		3192 sh	3123
		3171 str	3073
$\delta(NH_2)$	1595	1594	1606
	1565	1583	1572 str
$\delta(NH_2)$-H-bonded	1523	1549	1555 sh
		1530	
$\delta(H\text{-}C\text{-}(NH_2)_t)$	1281	1247	1240
	1257	1226	1172
	1228	1208	1130
	1199	1179	
	1169	1163	
	1148	1151	
ν(C-N)	1026	1072	1000
	1005	1052	976
	960	1046	982
$\delta(NH_2)$ w (out of plane)	911	938	966
	875	867	887
	822	799	821
ν(Pt-N)	-----	518	539, str.
		481	
		435	
ν(Pt-I)	-----	307	-----
		275	
		250	
$\nu(CO_3)$		-----	1451
			1410
			1162
			1131
			905
			871
			688
			618

br = broad; sh = shoulder; str = strong; w = weak

3. Solid-state NMR spectra

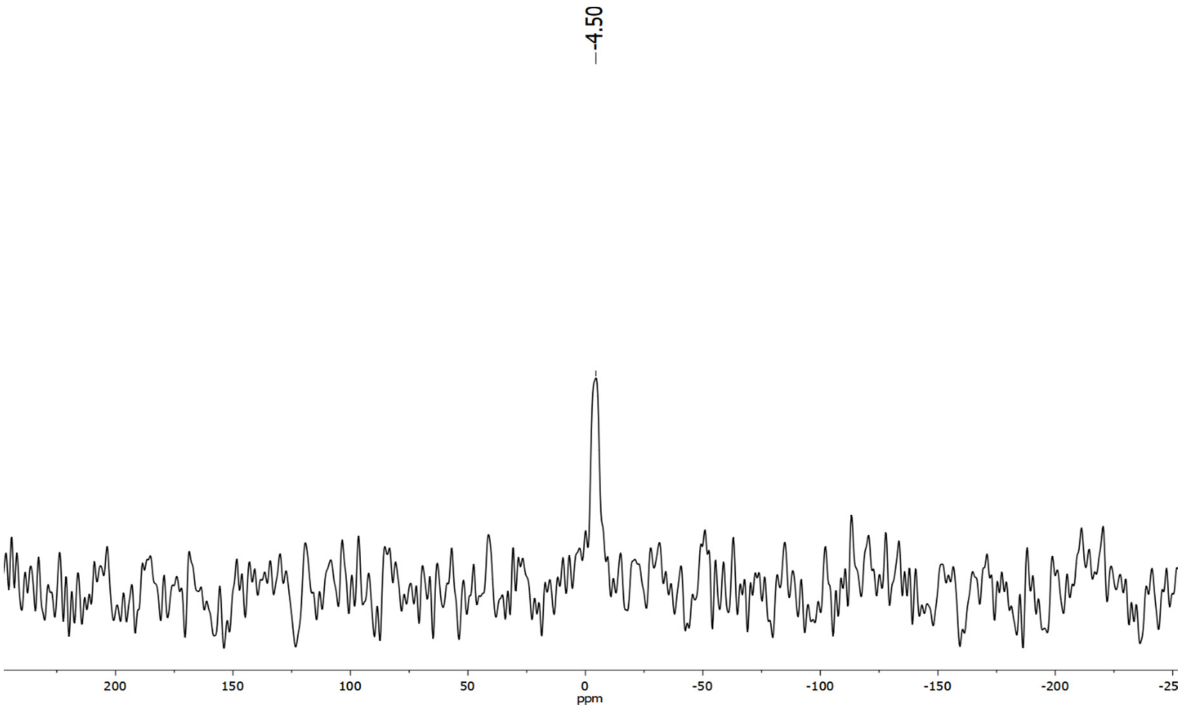

Figure S4. ^{15}N CP-MAS NMR spectrum of **1A**

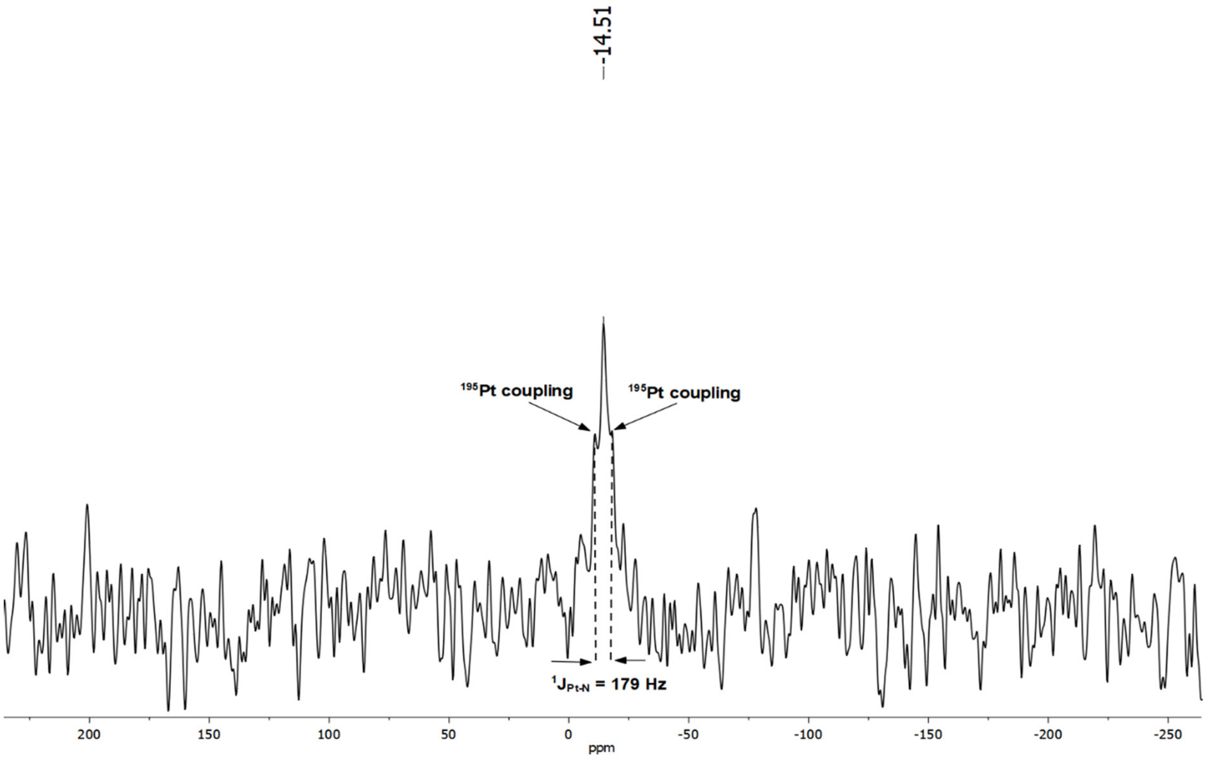

Figure S5. ^{15}N CP-MAS NMR spectrum of **2A.**

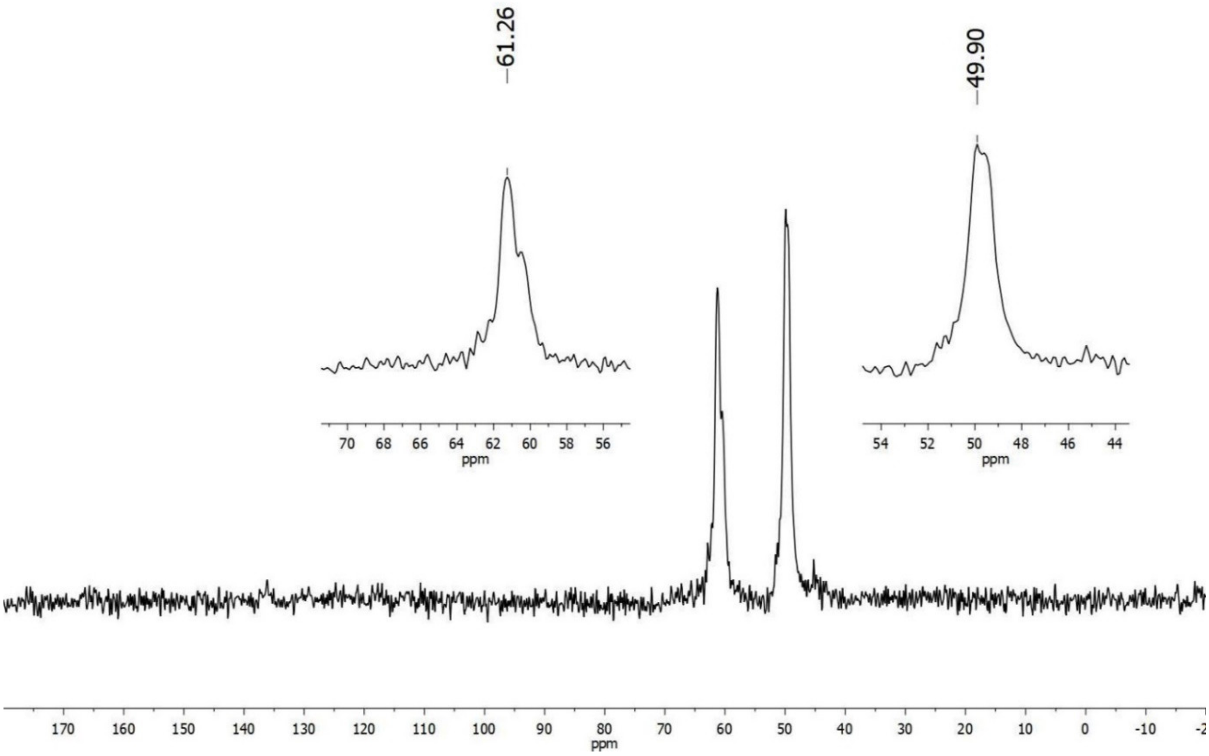

Figure S6. ^{13}CP-MAS NMR spectrum of **1A.**

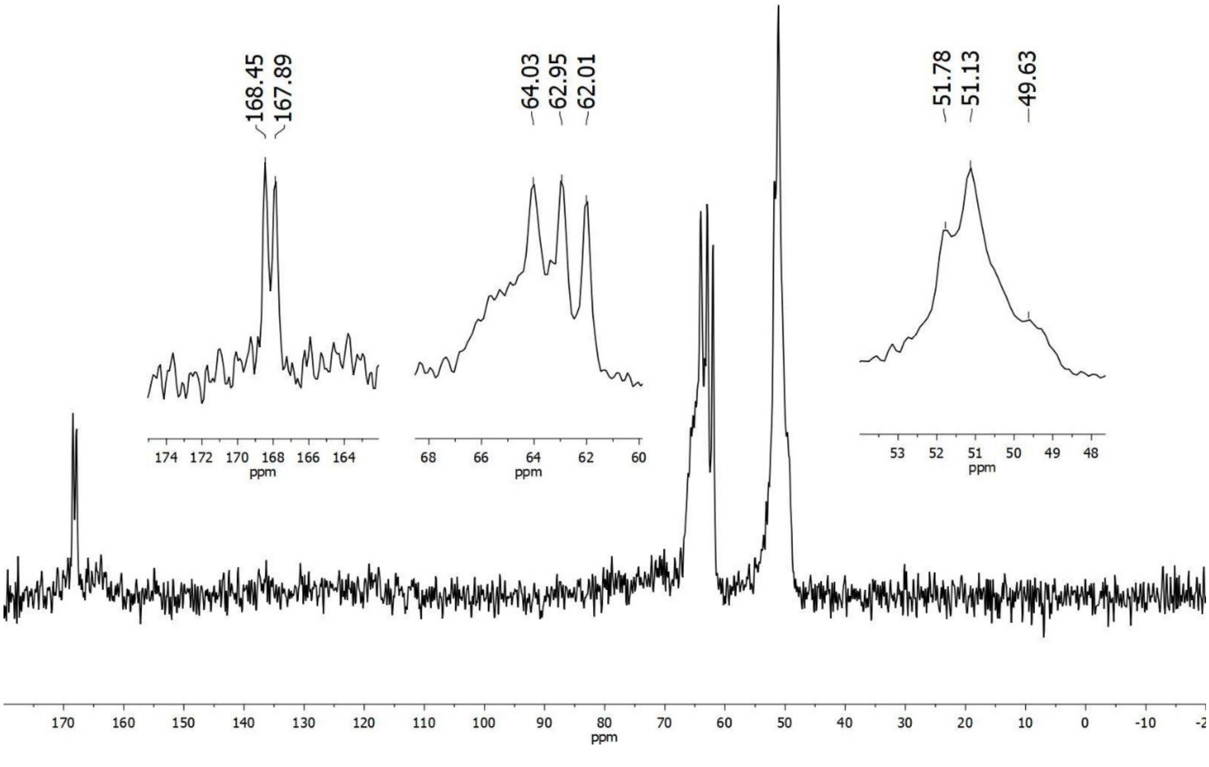

Figure S7. ^{13}C CP-MAS NMR spectrum of **2A.**

4. NMR Spectra (in solution).

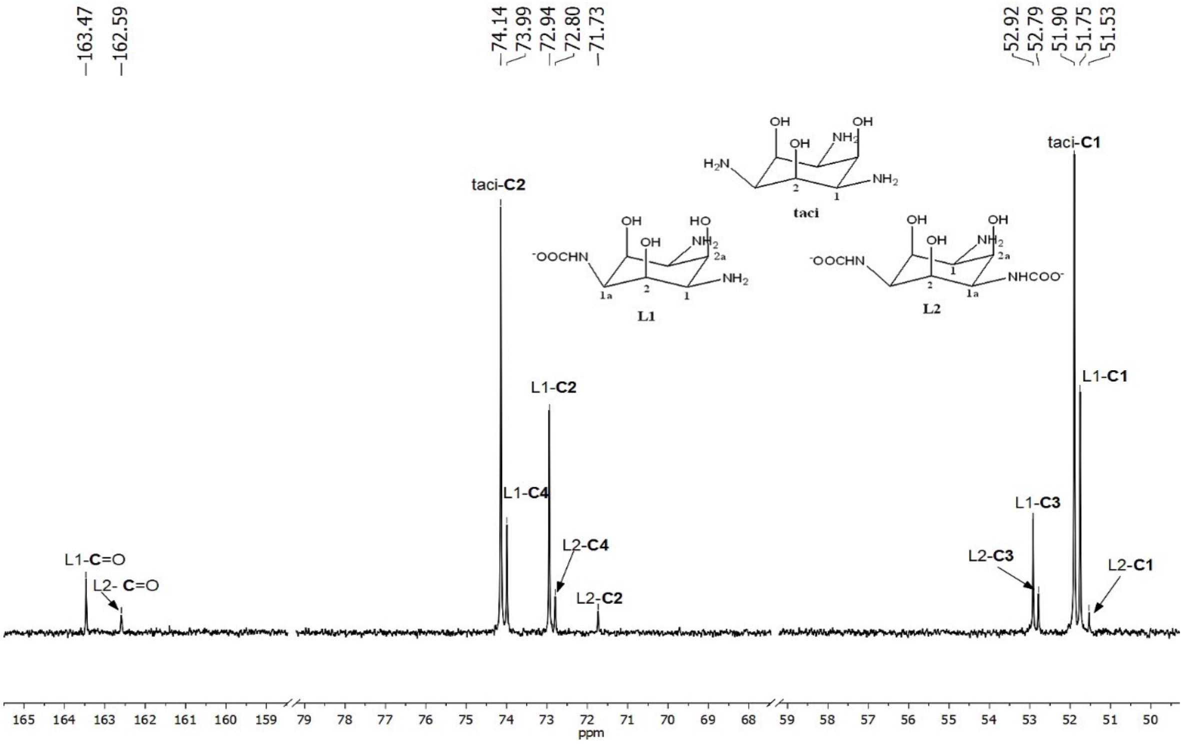

Figure S8. ^{13}C-NMR spectrum of taci.$2H_2O$ in D_2O, pD=8.4.

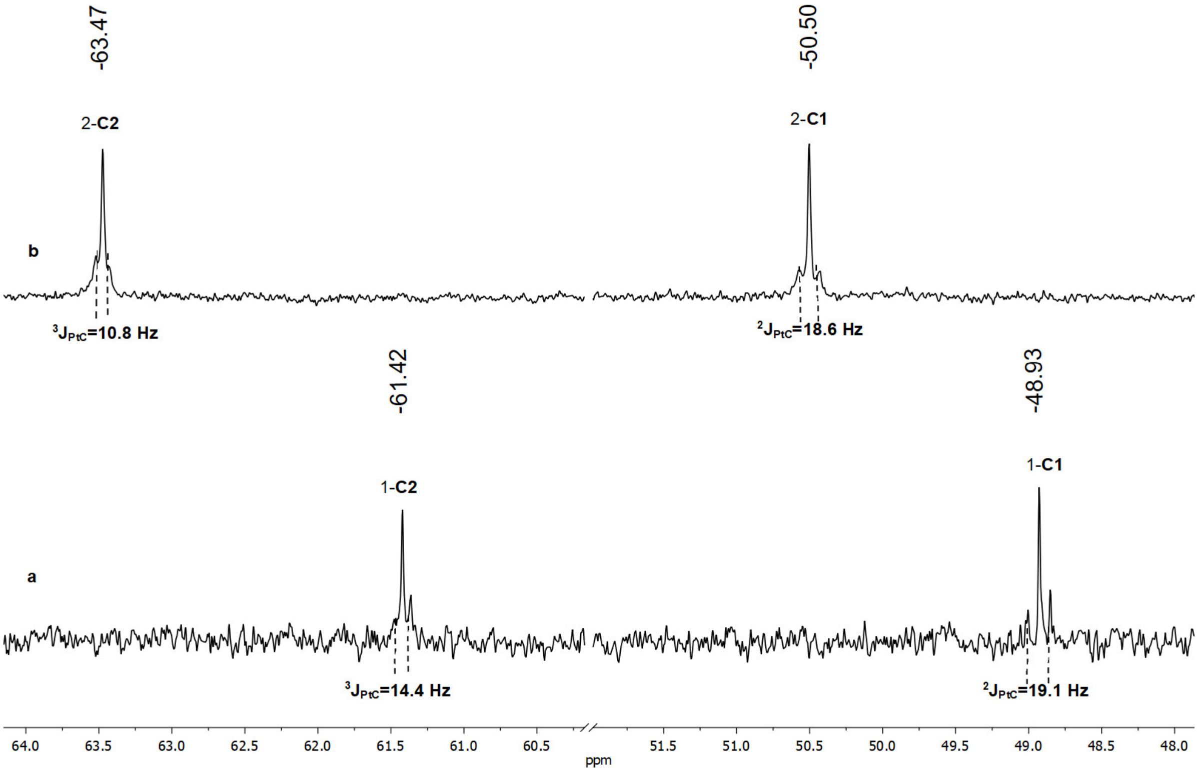

Figure S9. ^{13}C-NMR spectrum of **1** (a) and **2** (b) in D_2O.
